# Supplementary material for: New data on Beurlenia araripensis Martins-Neto & Mezzalira, 1991, a lacustrine shrimp from Crato Formation, and its morphological variations based on the shape and the number of rostral spines
Source: PLoS One. 2021 Mar 17;16(3):e0247497. doi: 10.1371/journal.pone.0247497 (PMC7968632; doi:10.1371/journal.pone.0247497)
Supplement: Attachment — Submitted filename: Response to Reviewers.docx [file pone.0247497.s003.docx]

**Response to Reviewers**

We thank the Reviewers for the considerations that have made a great contribution to the improvement of our work.

All grammatical changes in the text were made, as well as the exclusions of phrases suggested by the reviewer.

**Reviewers' comments:** Use open nomenclature, i.e. no taxonomic categories

**Answers:** The suggested replacement was made.

**Reviewers' comments:** Include a reference to the stratigraphy here.

**Answers:** It was addition to the text the reference Valença et al. [9]

**Reviewers' comments:** Slender than what?

**Answers:** It has been substituted for “Exopodite is smaller than ischium and merus”
